# Behavior disorders and subjective burden among caregivers of demented patients

**DOI:** 10.1590/s1980-57642008dn10200012

**Published:** 2007

**Authors:** Lucena Ferretti Ceres Eloah, Paulo Henrique Ferreira Bertolucci, Thaís Soares Cianciarullo Minett

**Affiliations:** 1 RN, Ms in Neuroscience, PhD in Science, Professor of Nursing College of Federal University of São Paulo.; 2 Neurologist, PhD, Professor of Neurology, Leader of Behavioral Neurology Unit of Federal University of São Paulo.; 3 Neurologist, PhD, Affiliated Professor of Federal University of São Paulo.

**Keywords:** dementia, behavioral disorders, caregiver distress, nursing intervention

## Abstract

Many patients with dementia live within the community supported by their family and friends. Majority of patient caregivers suffer from high levels of stress.

**Methods:**

A quasi-experimental study, which compared the effects of nursing interventions on behavior disorders in both patients with moderate to severe dementia and their caregivers, followed over 18 months. The assessments were performed at the outpatient clinic of the Federal University of São Paulo and again at patients’ homes with their primary caregiver, after informed consent form. Measurements were performed at baseline and after 18 months (pre and post-test). The instruments used were: The CDR, NPI and NPI-D; Katz Index and FAQ. Simultaneously, caregivers were enrolled onto the Dementia Education Program.

**Results:**

The final sample was composed of 31 subjects, having a mean age of 77.4 y.o. (±8 SD). Nursing interventions were effective in reducing some of the behavioral disturbances (Z= –3.1; p=0.002), such as Aggression (Z= –3.7; p<0.001) and anxiety (Z= –2.3; p=0.023). Caregiver distress also reduced upon interventions (Z= –2.2; p=0.030).

**Conclusion:**

Our results indicate nursing interventions may be effective in reducing the frequency and severity of behavioral disorders and subjective burden among caregivers. Education Programs can improve caregiver burden through conveying information on difficulties related to the disease and how to deal with them.

## Abstract (pt)

Muitos pacientes com demência vivem na comunidade amparados por suas famílias e amigos. Muitos destes cuidadores sofrem de altos níveis de estresse.

**Métodos:**

Estudo quase-experimental, que comparou os efeitos de intervenções de enfermagem nas alterações de comportamento de pacientes com demência moderada a grave, e seus cuidadores, acompanhados por 18 meses. As avaliações iniciais foram feitas no ambulatório de Neurologia do Comportamento da Universidade Federal de São Paulo e as subseqüentes no domicílio do paciente, com o cuidador primário, após consentimento informado. As avaliações foram realizadas na visita inicial e após 18 meses (pré e pós teste). Os instrumentos utilizados foram: CDR, INP e INP-E; Índice de Katz e QAF. Simultâneamente, os cuidadores foram incluídos no Programa de Educação em Demências.

**Resultados:**

A amostra final foi composta 31 sujeitos, com idade média de 77,4 (±8 DP). As intervenções de enfermagem foram efetivas na redução de algumas alterações de comportamento (Z= –3,1; p=0,002), como agressividades (Z= –3,7; p<0,001) e ansiedade (Z= –2,3; p=0,023). O estresse do cuidador também foi reduzido (Z= –2,2; p=0,030).

**Conclusão:**

As intervenções de enfermagem podem ser efetivas na redução da severidade e da freqüência das alterações de comportamento e na sobrecarga subjetiva entre cuidadores. Programas de Educação podem melhorar a sobrecarga dos cuidadores, por oferecer informações acerca das dificuldades relacionadas à doença e como lidarem com elas.

Many patients with dementia live within the community supported by their family and friends. Majority patient caregivers suffer from high levels of stress and depression.^1-3^ Behavior disorders such as irritability, aggressiveness, anxiety and depression, among others, are a common source of stress to caregivers. The most important determinant factor for the institutionalization of the patient is, most often, caregiver psychological morbity.^4^ Great economic, social and human efforts may be required to relieve this burden. Some studies have previously demonstrated the benefits of intervention to caregivers, in terms of improving psychological impact and of delaying institutionalization of patients.^5,6^ The development of support models based on guidance directed to these patients’ families seem to be a consensus in the literature.^7-9^

The pharmacological approach is efficient in treating non cognitive symptoms of dementia however, the side effects may limit its application. The use of neuroleptics commonly leads to parkinsonism, cognitive decline, constipation, urinary retention, among othersymptoms.^10, 11^ The ageing process increases the risk of these side effects - 50% of the subjects over 65 years old are prone to these.^12^ Depending on the type of dementia, this risk can be even greater. More than 80% of patients with Lewy Body Dementia are sensitive to typical neuroleptic standard doses, resulting in increased mortality.^13^ Other pharmacological groups, such as antidepressants, anxyolitics and sleep inducers, can lead to sleep disturbances, blurred vision, falls, gastrointestinal disturbances, tremor, rash, bradycardia, hypotension, bradypnea or apnea.

Another group of drugs, the cholinesterase inhibitors (AChEIs), has demonstrated improvement in cognition, function and behavior.^14-17^ The most common side effects of these drugs are gastrointestinal disturbances. However, these effects seem to be transitory. This is why the family needs to be informed on the benefits brought about by AChEIs treatment.

Concerning social and economic aspects, it is important to consider the high cost of these drugs.

A number of support groups have been created worldwide in a bid to delay institutionalization of patients, clarify doubts related to dementia diagnosis and treatment, and to manage patients in terms of function and behavior.^5,18-23^

## Hypothesis

Our hypothesis holds that non-pharmacological interventions can minimize the frequency and severity of behavioral disorders in dementia, while also reducing caregiver distress.

## Objectives

- To identify the impact of nursing interventions on behavior disorders in dementia,
- To verify whether these interventions have a positive impact on the patient - family - environment, and also, whether they reduce the frequency and severity of behavioral disturbances, thereby minimizing both use of drugs and caregiver burden.

## Methods

A quasi-experimental study, which compared the effects of nursing interventions on behavior disorders in patients with moderate to severe dementia, along with their caregivers, followed up at 18 months. The study was developed at the Behavioral Neurology Unit of the Federal University of São Paulo. Our sample comprised patients and their caregivers followed regularly at this service.

The inclusion criteria were: patients living in the community with an unpaid caregiver; dementia diagnosis according to the Diagnostic and Statistical Manual of Mental Disorders^24^ (DSM-IV) criteria, and presence of behavioral disorders.

The initial sample comprised 39 outpatients and their caregivers. All caregivers agreed to participate in this study and signed the Informed Consent Form at the time of enrollment. This study was approved by the Local Ethics Committee.

Both patient and caregiver assessments were carried out by a nurse with expertise in Neuroscience, in two different settings: first assessment at the behavioral neurology outpatients unit, and the subsequent assessment at the patients’ homes, with their primary caregiver. All caregivers lived with the patients.

Measurements were performed at baseline at the outpatient clinic, and after 18 months at patients’ homes (pre and post-test). The severity of dementia was determined by the Clinical Dementia Rating Scale (CDR), adapted to the Brazilian population.^25^ Frequency and severity of behavior disorders were measured using the Neuropsychiatric Inventory (NPI), while caregiver distress employed the Neuropsychiatric Inventory - Distress (NPI-D). ^26^ In addition, the activity of daily living and instrumental activity of daily living was assessed by the Katz Index^27^ and the Functional Activities Questionnaire^28^ (FAQ), respectively. After 18 months, the patients were submitted to a second full assessment. The instruments above were used in both assessment settings: outpatient clinic and homes.

Upon inclusion of the case in the study, caregivers were enrolled onto the Education Program on Dementia. The Dementia Education Program and Caregiver Assistance Program have both been developed by the Behavioral Neurology Unit at the Federal University of São Paulo. The main objectives of these programs are to offer preventative interventions.

It is important to provide information to caregivers on dementia progression, drugs and their side effects, as well as on management of activities of daily living in which patients are dependent, such as: eating, hydration, dressing, bladder and bowel continence, constipation, personal hygiene, and prevention of urinary and pulmonary infections, dehydration and skin ulcers. Indeed, it is also clear that caregivers sometimes need to receive psychological support and thus, whenever necessary, they were assessed by our psychologist from the behavioral unit.

Concerning the Education Program, the approach to the caregiver was based on the American Academy of Neurology guidelines, as follows^29^:

Interventions directed to the Patient:

- Bladder training to prevent and control urinary incontinence;
- Development of a day-to-day routine to promote self-care;
- Positive reinforcement to improve functional independence;
- Inclusion of the patient in simple instrumental activities with orientation and supervision;
- Soft music, mostly during bath and meal times;
- Physical activities, such as regular walking;
- Getting the patient to watch old movies on TV and view family photo albums;
- Use of simple commands at the patients comprehension level;
- Adequate lighting.
- Interventions directed to the caregiver:
- Education Programs in dementia – where they received educational orientation on the dementia process and care with the patient;
- Psycho-social Support Group;
- Inclusion of the family in the care plan – reinforcement on sharing tasks with other family members, using respite care when possible.

Following this first assessment patients were visited monthly at home. The aim of these visits was to identify factors that could be contributing to patients’ behavioral disorders. After identifying these factors, the nursing interventions were implemented, as follows:

1. Environment adaptations: such as improvement of the patients’ environment, suggestions regarding the floor, stairs, lighting, furniture, toxic materials and dangerous objects, among others that could cause personal accidents.
2. Training on coping techniques for behavior disorders, and feeling of losing quality of life related to the dementia progression. This assessment included explanation about the disease process.

- Caregiver education - Caregivers attended educational group meetings once a month lasting two hours, where they received manuals containing information about the disease and non-pharmacological behavioral management.

Whenever needed additional orientations were available over the telephone.

### Statistical analysis

Differences between continuous data means, before and after nursing interventions, were compared using the Wilcoxon Signed Rank (Z) test. Data obtained from the patients who were not submitted to the second assessment were not dropped. To indicate the statistical significance, a probability (p) of less than 0.05 was considered. All tests were bi-caudated.

The program used in statistical analysis was the Statistical Package for the Social Science - SPSS - for 11.5.1Windows version.

## Results

The patient sample was composed of 27 women (71%) and 11 men (29%). Patients’ mean age was 77.4 (±8 SD) and of the 39 patients included at the initial sample, 31 subjects were finally included, having completed the second assessment. One of these patients did not fulfill the inclusion criteria, while another 7 died due to pneumonia or urinary infections. Nobody was institutionalized and none of the subjects refused to participate.

Table 1 shows the descriptive analysis of the scales of scores in both assessments.

**Table 1:** Descriptive analysis of scores obtained on both evaluations.

		Mean	Standard deviation	Medium	Minimal	Maximum
NPI	1^st^ evaluation	43.7	18.9	47	6	76
	2^nd^ evaluation	35.5	20.8	36	6	88
NPI- D	1^st^ evaluation	18.6	8.2	18	0	38
	2^nd^ evaluation	16.1	10.4	15	0	50
KATZ index	1^st^ evaluation	1.3	1.4	1	0	6
	2^nd^ evaluation	1.6	1.3	1	0	4
FAQ	1^st^ evaluation	25.9	5.1	27	13	30
	2^nd^ evaluation	26.2	4.1	27	17	30

1^st^ evaluation: n=38; 2^nd^ evaluation: n=31.

### Second assessment

After a mean period of 18 months (±0.4SD), of the sample of 28 subjects on behavioral medications, 11 had discontinued them as their medical prescription. Three patients were not on this group of medications (behavioral disturbances control), and only one of subject began taking behavioral drugs. The other two patients belonging to this subgroup continued not to use these drugs.

### Response to nursing interventions

Comparison between the total score on the NPI in both assessments, showed a statistically significant improvement (Z= –3.1; p=0.002). NPI items such as, Aggression (Z= –3.7; p<0.001) and anxiety (Z= –2.3; p=0.023) also improved significantly. Caregiver distress also reduced with the interventions (Z= –2.2; p=0.030) as measured by the NPI-D.

Instrumental Activities of Daily Living before and after intervention did not differ significantly (Z= –0.7; p=0.507), while scores on the KATZ Index showed a statistically significant improvement in the activities of daily living (Z= –2.4; p=0.014) (Table 2).

**Table 2:** Comparison of total NPI scores and their subitems; KATZ INDEX; FAQ, and caregiver stress before and after nursing interventions.

	Before interventions	After Interventions	Z	p
Aggressiveness	8.6	5.7	-3.7	<0,001
Hallucinations	2.7	2.5	-0.4	0.689
Anxiety	4.6	3.6	-2.3	0.023
Apathy	1.8	1.6	-0.2	0.858
Eating	1.5	2.5	-2.0	0.050
Aberrant motor behavior	3.4	2.1	-1.8	0.074
Delirium	5.7	4.5	-1.9	0.063
Disinhibition	2.1	2.1	0.0	0.969
Dysphoria	1.6	0.7	-2.2	0.026
Euphoria	0.4	0.3	-0.6	0.564
Irritability	8.9	7.5	-1.8	0.065
Sleep	2.6	2.3	-0.7	0.454
NPI- D	18.7	16.1	-2.2	0.030
KATZ index	1.2	1.6	-2.4	0.014
FAQ	26.4	26.2	-0.7	0.507
NPI	43.9	35.5	-3.1	0.002

N=31

## Discussion

The present study hypothesis may be confirmed by the reduction of caregiver burden levels, by the score reductions on some items of the NPI scale and, additionally by the functional improvement of Activities of Daily Living. However, there was no improvement in the Instrumental Activity of Daily Living performance. In fact, the patients performance for IADLs had worsened over the 18-month period, which can possibly be explained by the dementia course, where patients’ skills requiring executive and planning functions, became progressively impaired, such as the ability to prepare foods, pay bills, housekeeping and so on.

Our main hypothesis was that through home visits and periodic meetings with caregivers, it would be possible for nurses to reduce the frequency of behavior disorders, and even improve the objective and the subjective burden of the caregivers. We have found similar models of before and after, non-pharmacological interventions in the literature that have involved education and support. Similar studies also aimed to reduce the subjective burden among caregivers.^8,30-32^ These studies, among other issues, hold that this kind of intervention, involving support to the caregivers through education, can be very effective. However, comparisons between the various studies are hampered due to methodological differences, a point raised in a review of 69 papers discussing support given to caregivers.^7^ These authors emphasized the importance that support interventions may have on the assistance, educational and guidance aspects - a finding compatible with the results of our study, which also suggests that educational programs in dementia can be effective.^9,33^

In dementia, the objective of family-oriented intervention is to reduce the negative sensation of caregivers’ feeling of burden, rather than any other direct intervention on the disease. Even so, the principles of the interventions remain the same. These interventions can be positive in the early phases of the disease, as has been demonstrated in a study similar to ours which used combined therapy in patients with initial to moderate Alzheimer’s disease and their families.^34^ This study showed great improvement in anxiety and depressive symptoms among caregivers achieved through interventions of support and counseling to these caregivers. Our findings have shown that combined therapy can also be useful in moderate to late stages of dementia.

Behavior disorders are expected during the course of dementia. The lack of information on how to manage these adds great burden to caregivers. Caregivers’ stress may arise from objective burden - related to direct care of the patient - or from subjective burden - related to caregivers’ perception of the problem and the feeling concerning this. This is a complex process which may vary according to patient and caregiver characteristics. The way caregivers react to the situation can determine how the patient will be managed.^35,36^

Although the results of the present study suggest that non-pharmacological interventions may reduce the frequency and severity of the behavioral disorders and subjective burden among caregivers, a lack of a control group represented the main limitation of this study, since comparison between two groups (with and without intervention) could reinforce the positive findings presented in the current study. Future case-controlled studies involving greater samples are needed to further verify our findings.

Although we have seen positive results from our interventions, we have also observed some side effects. For some subjects, a negative impact of the education program was observed, but mostly related to the format itself, such as: high volume of information during meetings; heterogeneous caregiver groups, whereby some needed more basic information whereas others needed deeper knowledge; difficulty in attending meetings related to transport, or lack of another caregiver to take care of the patient while they were away. Another obstacle was the belief, expressed by the caregiver, that they could not attend the meetings because nobody would be able to care for their patients as well as they could.

## Conclusion

Nursing intervention may be effective in reducing both frequency and severity of behavioral disorders and subjective burden among caregivers. Education Programs for caregivers can develop a greater sense of competence and self-confidence in such individuals, when coping with the difficulties related to the management of dementia. Besides, this approach can promote and improve caregiver adherence to pharmacological and non-pharmacological treatment. Finally, it can also result in quality of life and long term reduction of costs to the health system through the reduction of comorbities common in these patients.
